# Mitigating excessive heat in Arabica coffee using nanosilicon and seaweed extract to enhance element homeostasis and photosynthetic recovery

**DOI:** 10.1186/s12870-024-05784-0

**Published:** 2024-11-12

**Authors:** Ekkachak Chandon, Patchawee Nualkhao, Metee Vibulkeaw, Rujira Tisarum, Thapanee Samphumphuang, Jianqiang Sun, Suriyan Cha-um, Suravoot Yooyongwech

**Affiliations:** 1 School of Interdisciplinary Studies (Kanchanaburi Campus), Mahidol University, Kanchanaburi, 71150 Thailand; 2 National Center for Genetic Engineering and Biotechnology (BIOTEC), National Science and Technology Development Agency (NSTDA), Pathum Thani, 12120 Thailand; 3 Research Center for Agricultural Information Technology, National Agriculture and Food Research Organization, 3-1-1 Kannondai, Tsukuba, Ibaraki 305-8517 Japan

**Keywords:** Arabica coffee, Chlorophyll content, High temperature, Photosynthetic efficiency, Silicon-magnesium homeostasis

## Abstract

**Background:**

Global warming-related temperature increases have a substantial effect on plant and human health. The Arabica coffee plant is susceptible to growing in many places across the world where temperatures are rising. This study examines how nanosilicon and seaweed extracts can improve Arabica coffee plant resilience during heat stress treatment (49.0 ± 0.3 °C) by maintaining mineral homeostasis and photosynthetic ability upon recovery.

**Results:**

The principal component analysis arrangement of four treatments, nanosilicon (Si), seaweed extract (SWE), Si + SWE, and control (CT), showed each element ratio of magnesium, phosphorus, chloride, potassium, manganese, iron, copper, and zinc per silicon in ambient temperature and heat stress that found influenced upper shoot rather than basal shoot and root within 74.4% of largest feasible variance as first principal component. Magnesium and iron were clustered within the silicon group, with magnesium dominating and leading to a significant increase (*p* ≤ 0.05) in magnesium-to-silicon ratio in the upper shoot under heat conditions, especially in Si and Si + SWE treated plants (1.11 and 1.29 fold over SWE treated plant, respectively). The SWE and Si + SWE treated plants preserved chlorophyll content (15.01% and 28.67% over Si-treated plant, respectively) under heat stress, while the Si and Si + SWE treated plants restored photosynthetic efficiency (F_v_/F_m_) better than the SWE treated plant.

**Conclusions:**

The concomitant of the Si + SWE treatment synergistically protected photosynthetic pigments and F_v_/F_m_ by adjusting the magnesium-silicon homeostasis perspective in Arabica coffee to protect real-world agricultural practices and coffee cultivation under climate change scenarios.

**Supplementary Information:**

The online version contains supplementary material available at 10.1186/s12870-024-05784-0.

## Background

Unquestionably, extreme climate change affects natural and agricultural ecosystems, crop species, and physiological mechanisms [1, 2]. Coffee, a globally important economic plant, is extremely sensitive to high temperatures [3]. Arabica coffee yields drop significantly in hot weather when high temperatures inhibit photosynthesis, affecting Arabica coffee growth and yield [3, 4]. In the current global environment, excessive temperatures may reduce coffee production areas [5]. Coffee plantations must adapt to specific farming and socioeconomic conditions [6]. Most coffee growers are smallholders; therefore, technology access must be considered [6]. Pham et al. [7] also recommended raising awareness and providing technical assistance to help farmers adapt to climate fluctuations and change. A suitable exogenous treatment can also increase resilience and heat stress resistance across a wide range of plant cultivation dimensions [8].

Exogenous treatment improves plant processes, as evidenced by research conducted under abiotic stress conditions, including heat [9]. Silicon has been shown to affect physiological features and biochemical links that increase plant resilience to severe abiotic stressors [10, 11]. Silicon regulation, which produces heat and drought conditions, can increase transpiration, stomatal behaviour, water loss, photosynthesis, and plant development [12, 13]. Silicon nanoparticles, improved leaf water retention and membrane stability in *Triticum aestivum* under 45 °C heat stress [12]. In addition, the silicon application indicated that root absorption could increase element availability, but many questions remain [14]. Understanding how the environment reacts to stress and the complex interactions between silicon and plant species is crucial [13]. Furthermore, it is also effective for seaweed extract, demonstrating their diverse roles and complex interactions in plant growth and development, including offering a resource for ecologically sustainable agriculture [15–17]. Seaweed extract acting as an exogenous treatment could be able to alleviate the harmful effects caused by nutrient deficiencies [18]. Seaweed extractmay act as a biostimulant given its ability to assist maize, tomato, lettuce, and almonds in absorbing iron (Fe), zinc (Zn), manganese (Mn), potassium (K), and magnesium (Mg) [19]. Extract of *Ascophyllum nodosum* seaweed alleviates heat stress (30 °C) and improves vigor in spinach seedlings compared to control (15 °C) [20].

Furthermore, the significance of mineral homeostasis in photosynthetic pigment interaction and efficiency is concerning. Plants need Mg, an essential micro-element, for physiological and metabolic activities, growth, and stress resistance [21]. Photosynthesis in plants requires Mg^2+^, which is the central Mg-atom structure of the chlorophyll molecule [22]. About 15–35% of transferred Mg modulates chlorophyll pigment homeostasis [23]. Additionally, an appropriate supply of nutritional ion balance in chloroplasts, such as potassium (K), phosphorus (P), and chloride (Cl), or copper (Cu), iron (Fe), manganese (Mn), and zinc (Zn), could lead to ion homeostasis, which is representative of the photosynthetic system [24–27]. This study hypothesizes that Si and SWE will improve the excessive heat tolerance in Arabica coffee by maintaining mineral homeostasis and photosynthetic efficiency. The primary objective of this study was to restore element homeostasis (Mg, P, Cl, K, Mn, Fe, Cu, and Zn) in root, lower, and upper shoot sections of the Arabica coffee plant after heat (49 °C) disturbance, as well as to locate variations in photosynthetic performance relating to coffee plant resilience.

## Methods

### Plant materials, treatments, and experimental setup

Nine-month-old seedlings of commercial Arabica coffee (*Coffea arabica* L. cultivar “Chiang Mai 80”) were procured from the Chiang Mai Royal Agricultural Research Center, Chiang Mai, Thailand. Forty coffee plants were grown in a 4 × 8 inch plastic bag containing soil substrates (EC = 2.687 dS m^-1^; pH = 5.5; organic matter = 10.36%; total nitrogen = 0.17%; total phosphorus = 0.07%; total potassium = 1.19%) under ambient greenhouse conditions with 50% shading to prevent leaf burn. Each plant received 250 mL of irrigation water every day from 8.00 to 9.00 am. Slow-release fertilizer (Osmocoat^®^ 13-13-13; N-P-K) was applied at a rate of 5 g per plant the first year of the Arabica coffee plant, as recommended by the Department of Agriculture (DOA), Thailand. The study used a completely randomized design (CRD) with four treatments: untreated control (CT), nano-silicon (nSiO_2_: Si), seaweed extract (SWE), and a combination of Si and SWE treatment (Si + SWE). The plants were arranged in 4 × 2 factorials at two different temperatures: ambient (32 ± 2 °C; Am) and heat stress (49 ± 0.3 °C; Ht), with three replications (*n* = 3) for element analysis and four replications (*n* = 4) in photosynthetic relating and growth performance.

The nSiO_2_ used in the study was supplied by Sigma-Aldrich (St. Louis, MO, USA) and has a product surface area of 175–225 m^2^ g^-1^ and a diameter of 12 nm. The effective Si concentration of 4 mM was used in accordance with Mustafa et al. [28]. The SWE from *Ascophyllum nodosum* commercial extract was supplied by Phytotech and Agrochemical Supplier, Chiang Mai, and verified by the Department of Business Development, Ministry of Commerce, Thailand (SWE details in Supplementary Table S1). The SWE was dissolved in water at 0.4% (w/v), according to Noli et al. [29]. Si + SWE was prepared by dissolving Si and SWE in water and mixing them in the same concentration. Soil drenching treatments with Si, SWE, Si + SWE, and CT (water) were applied to five plants on days 0 and 6.

Fourteen days later, the plants were subjected to heat stress at 49 ± 0.3 °C for 50 min [30] in a 200-liter hot-air oven (OV200, Kluay Nam Thai Trading Group Co., Ltd., Bangkok, Thailand). with airflow of 0.71 ± 0.04 m s^-1^ and RH of 51.75 ± 1.37%. The plants were examined at 32 ± 2 °C, airflow 0.45 ± 0.05 m s^-1^ and 76.81 ± 1.02% RH for ambient conditions. The photosynthetic efficiency (maximum quantum yield; F_v_/F_m_) was assessed after 30 min and 24 h of heat recovery. After the 24-hour heat explosion recovery time, plant samples were processed and analysed for study.

### Element analysis

The shoot apex, basal shoot (4 cm from the apex or basal), and root tissues were collected and dried at 60 °C in the hot air oven (FED115, Binder, Tuttlingen, Germany) until the weight remained constant. Plant samples were ground in a mortar with liquid nitrogen into a powder (0.5 g) and kept in the desiccator. Prior to examination, the powder was compacted under a 37 mm diameter and 2 mm height sample load holder. The sample is then loaded into a PANalytical Zetium PW5400 wavelength dispersive X-ray fluorescence (WD-XRF) spectrometer (Malvern Panalytical, Malvern, UK) for element measurement. XRF sample preparation was straightforward with non-acid digestion and accurate measurement [31]. The Malvern Panalytical SuperQ program was used to express element concentration sensitivity (%). The element content was converted into an elemental ratio by Si, each element (g kg^-1^) per Si (g kg^-1^) using the formula given by Yatkin et al. [32]. The mineral translocation factor (TF) from root to shoot was calculated as the ratio of element concentration (mg kg^-1^) in the apex- or basal-shoot to the root tissues, according to the modified method given by Prabasiwi et al. [33] and Vera Tome et al. [34].

### Photosynthetic efficiency (F_v_/F_m_) measurement

The maximum quantum yield (F_v_/F_m_) was estimated using chlorophyll fluorescence, a sensitive indicator of photosynthetic ability [35]. The F_v_/F_m_, equal to the (F_m_−F_0_)/F_m_, was measured from the second to third mature fully expanded leaves [36] from the shoot apex using a Handy-PEA chlorophyll fluorometer (Hansatech, UK). In brief, the leaves were stimulated in the dark for 30 min using leaf clips. The F_v_/F_m_ of dark-adapted leaves, which corresponds to the minimal (F_0_) and maximal fluorescence yield (F_m_), was determined [37]. According to Lima-Moro et al. [38], a lower F_v_/F_m_ value is caused by higher PSII light inhibition (i.e., greater F_0_) and lower PSII electron transport rate (lower F_m_). To assess the photoinhibition and stress in the control plant, the relative relative F_v_/F_m_ recovery was calculated. The relative F_v_/F_m_ recovery was determined by comparing it to a control plant zero line under ambient conditions using the following equation (1).

$$\begin{aligned}&\text{R}\text{e}\text{l}\text{a}\text{t}\text{i}\text{v}\text{e}\:{\text{F}}_{\text{v}}/{\text{F}}_{\text{m}}\:\text{r}\text{e}\text{c}\text{o}\text{v}\text{e}\text{r}\text{y}\:\left(\%\right)\cr&=\left[\frac{\:{\text{F}}_{\text{v}}/{\text{F}}_{\text{m}\_\text{t}\text{n}\_\text{h}\text{e}\text{a}\text{t}}\:\text{o}\text{f}\:\text{t}\text{a}\text{r}\text{g}\text{e}\text{t}\:\text{p}\text{l}\text{a}\text{n}\text{t}\text{s}}{\text{M}\text{e}\text{a}\text{n}\:{\text{F}}_{\text{v}}/{\text{F}}_{\text{m}\_\text{t}0\_\text{A}\text{m}\text{b}\text{i}\text{e}\text{n}\text{t}}\:\text{o}\text{f}\:\text{c}\text{o}\text{n}\text{t}\text{r}\text{o}\text{l}\:\text{p}\text{l}\text{a}\text{n}\text{t}\text{s}}\times100\right]-100\end{aligned}$$

Where F_v_/F_m_tn_heat_ is of plants at time t_n_ after heat treatment, where t_n_ is 30 min and 24 h. The F_v_/F_m_t0_Ambient_ is F_v_/F_m_ in the control plants.

### Chlorophyll and carotenoid contents and growth performance

The second to third fully developed leaves from the shoot apex were collected to assay photosynthetic pigments. Fresh samples were thoroughly ground in a mortar and then extracted with 80% acetone, following the modified method described by Lichtenthaler [39]. The absorbance was measured at 470, 645, and 662 nm proper using a UV-visible spectrophotometer (DR/4000; Model 48000 HACH, Loveland, CO, USA). The acetone was used as a blank. Then, the concentration of chlorophyll *a*, chlorophyll *b*, total chlorophyll, and total carotenoids was calculated using the following equations, (2) to (5), respectively:

$$\text{Chlorophyll}\:a=\frac{\left[\right(12.25\text{OD}_{662}\:-\:2.79\text{OD}_{645})\:\times\:\text{V}]}{\left[\right(1000\:\times\:\:\text{W}\left)\right]}$$

$$\text{Chlorophyll}\:b=\frac{\left[\right(21.50\text{OD}_{645}-5.10\text{OD}_{662})\times\text{V}]}{\left[\right(1000\times\text{W}\left)\right]}$$

$$\text{Total}\text{Chlorophyll}\hspace{0.17em}=\hspace{0.17em}\text{Chlorophyll}\:a\hspace{0.17em}+\hspace{0.17em}\text{Chlorophyll}\:b$$

$$\begin{aligned}&\text{Total}\:\text{carotenoids}\:(Cx+c)\cr&\quad=\frac{\left[\frac{(1000\text{OD}_{470}-1.82\text{Chl}\:a-85.02\text{Chl}\:b)}{(198\times\text{V})}\right]}{\left[\right(1000\times\text{W}\left)\right]}\end{aligned}$$

where V is acetone volume and W is sample weight.

After the 24-h heat stress, shoot height was measured from shoot apex to basal shoot at soil level with a measuring tape. Upper (4 cm from shoot apex), lower (4 cm from basal shoot), and total shoot biomass were weighted and averaged. Plant height and the fresh biomass were measured in four treatments at both temperatures.

### Data curation and statistical analysis

The ratios of the eight elements (e.g., Mg, P, Cl, K, Mn, Fe, Cu, and Zn) per silicon and the element TF to coffee plant parts measured under ambient and heat conditions in the four treatments: Si, SWE, Si + SWE, and CT were directly input to Jamovi version 2.3.18 (https://www.jamovi.org) for principal component analysis (PCA). Data standardization was conducted before PCA automatically by the Jamovi software. A biplot of PCA was carried out to explain the influences derived from elements under ambient and heat conditions. A heatmap was auto-standardized and grouped by element content for the two scenarios. The heatmaps were hierarchically clustered with Ward’s method based on Euclidean distances. A clustering dendrogram and PCA ranked relationships F_v_/F_m_ (30 min and 24 h) recovery channels for CT, Si, SWE, and Si + SWE treatments. A Pearson correlation coefficient was used to direct line-pair scattering for the growth variables in linear connection, according to the growth study. Additionally, the analysis of variance (ANOVA) and mean ± standard error (SE) were calculated. The data were analysed by post hoc Duncan’s new multiple range test (DMRT) at *p* ≤ 0.05 conducted using R version 4.2.1.

## Results

### Element homeostasis

**Fig. 1:**
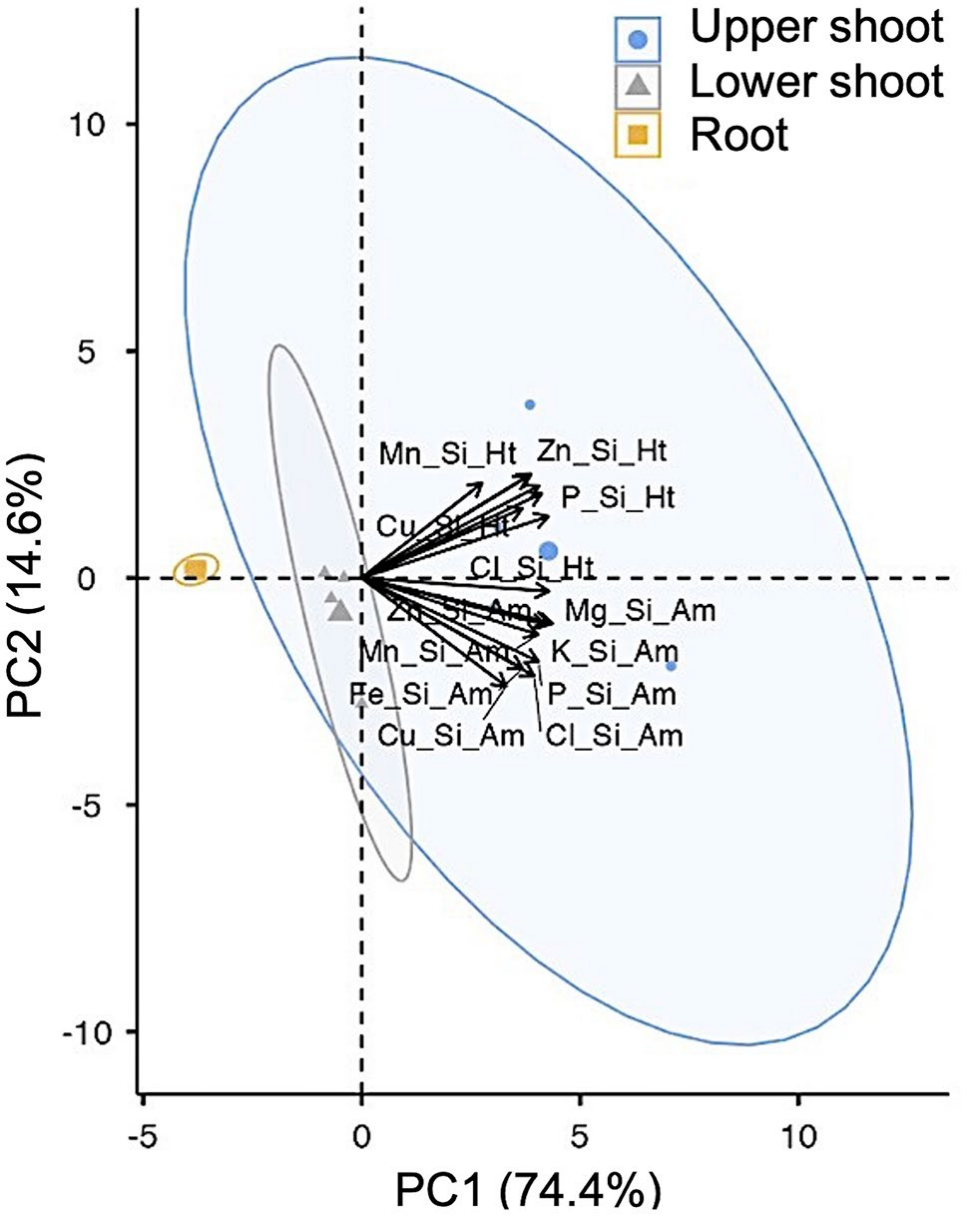
Group and vector plot of principal component analysis (PCA) in each element (magnesium, phosphorus, chloride, potassium, manganese, iron, copper, and zinc) ratio per silicon in the upper shoot, lower shoot, and root of the coffee plant under ambient (Am) and heat (Ht) conditions

**Fig. 2:**
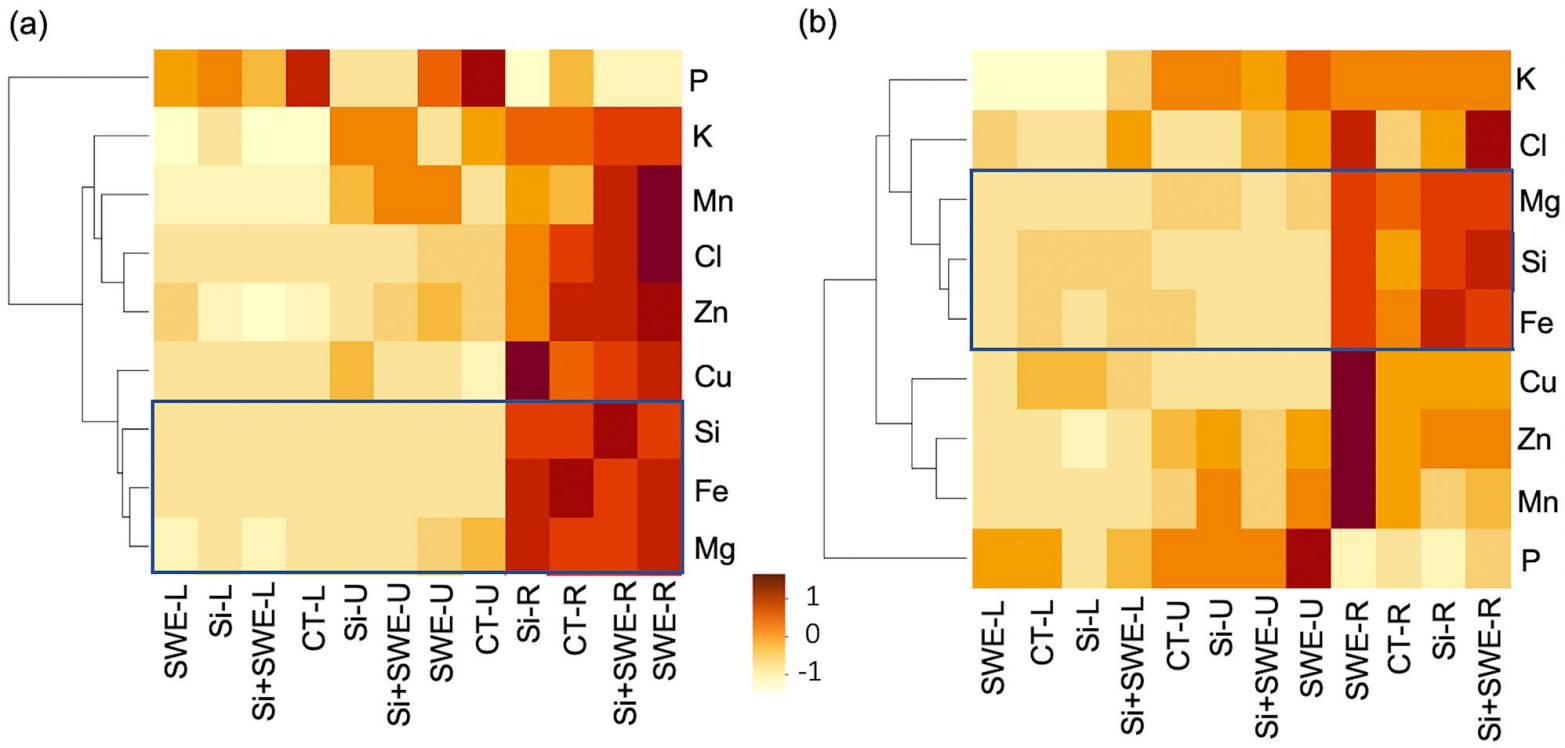
Cluster analysis with heat map dendrogram for study element (magnesium, phosphorus, chloride, potassium, manganese, iron, copper, zinc, and silicon) content in the upper shoot (U), lower shoot (L), and root (R) of the coffee plant in the control (CT), silicon (Si), seaweed extract (SWE), and Si plus SWE (Si + SWE) under the (**a**) ambient and (**b**) heat conditions. Euclidean distance clustering showed nine element contents in columns. Rows indicated twelve combinations of these four treatments and three plant parts. A gradient color scale indicated a relative maximum (1) and minimum (-1)

The Si-treated plants showed changes in elemental homeostasis, as indicated by the element-to-Si ratio (Fig. 1). Figure 1 shows the PCA of element ratios in 16 variations of the Mg, P, Cl, K, Mn, Fe, Cu, and Zn elements, along with the heat and ambient conditions, revealed a separation in an upper shoot zone in the Arabica coffee to 74.4% first principal component (PC1). The majority of these elements’ contents vary depending on the ambient temperature and heat conditions, especially in the upper and lower shoot portions (Fig. 2). Nevertheless, a hierarchical clustering result indicated It is remarkable that the Si concentration was highly significant correlated with Mg (correlation coefficient = 0.940 and 0.942) and Fe (0.950 and 0.973) in both ambient and heat conditions, respectively (Fig. 2, Supplementary Table S2). It suggests an interplay between Si, Mg, and Fe in response to element maintenance, even under different temperature conditions.

### Magnesium and iron-related silicon in response to heat treatment

**Fig. 3:**
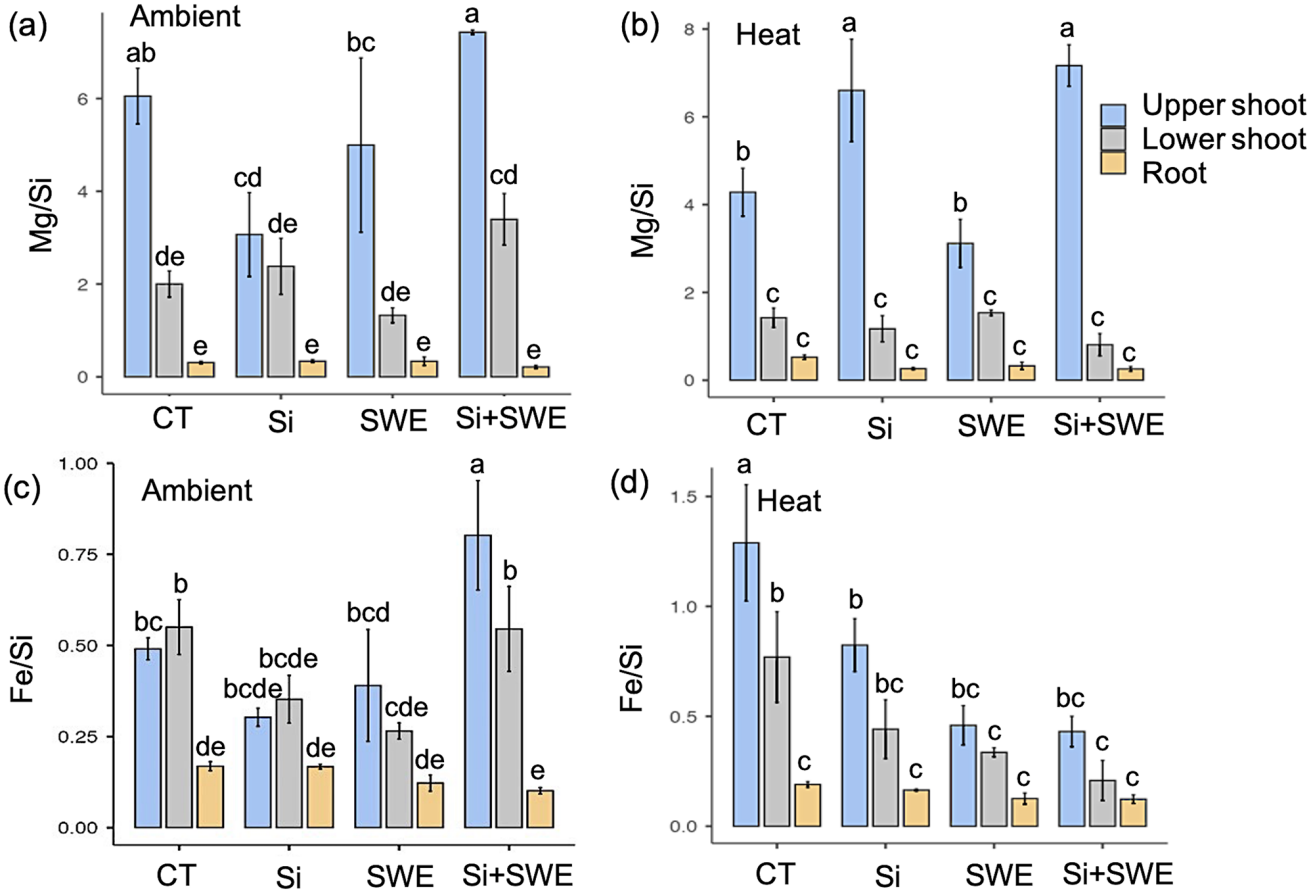
Comparison ratios of (**a**, **b**) magnesium per silicon, Mg/Si, and (**c**, **d**) iron per silicon, Fe/Si, in the upper shoot, lower shoot, and root of the coffee plant in the control (CT), silicon (Si), seaweed extract (SWE), and Si plus SWE (Si + SWE) under ambient and heat conditions. Different letters in each column represent significant differences (*p* ≤ 0.05) using DMRT

**Fig. 4:**
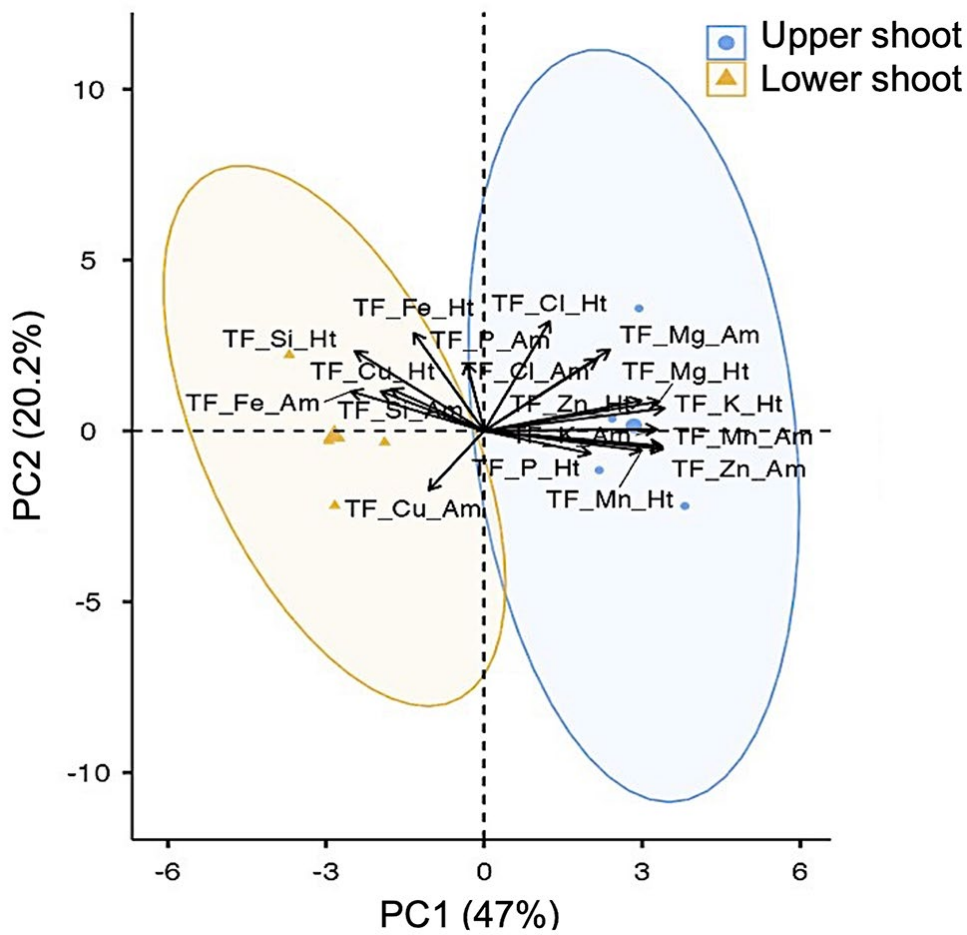
Principal component analysis (PCA) of element transferring factor (TF; ratio its self-element of magnesium, phosphorus, chloride, potassium, manganese, iron, copper, zinc, and silicon) from the root to the upper and lower shoots in the coffee plant under ambient (Am) and heat (Ht) conditions

Si influence suggests that Mg and Fe are involved in the group interaction (Figs. 1 and 2). Si induced Mg and Fe homeostasis. The Mg/Si ratio in the upper shoot of all four treatments ranged from 3.07 to 7.43 under both ambient and heat conditions, whereas it was decreased in basal shoot and root sections (Fig. 3a, b). In heat, the Mg/Si ratio in the upper shoot of the Si-treated and Si + SWE plants increased by 6.60 and 7.17, respectively, compared to the CT (4.28) and SWE plants (3.12) (Fig. 3b). The basal shoot and the roots in the four treatments under the heat had Mg/Si ratios of 0.25 to 1.53. In the basal shoot and the roots, this ratio significantly dropped by more than 50% compared to the upper shoot (Fig. 3b). Compared to CT plant, Si-treated, SWE, and Si + SWE plants in upper shoots had significantly lower Fe/Si in heat conditions (Fig. 3d). The Mg and Fe ratios improved only in the upper shoot of Si + SWE and CT plants under the ambient conditions (Fig. 3a, c). In addition to analyzing an element-transferring factor, the PCA revealed that Mg dominated movement into the upper shoot (from root to basal- and upper-shoot parts) in both ambient and heat environments, while Fe dominated movement from root to basal shoot, but not in the upper shoot (Fig. 4).

### Photosynthetic efficiency, photosynthetic pigments, and growth performance

**Fig. 5:**
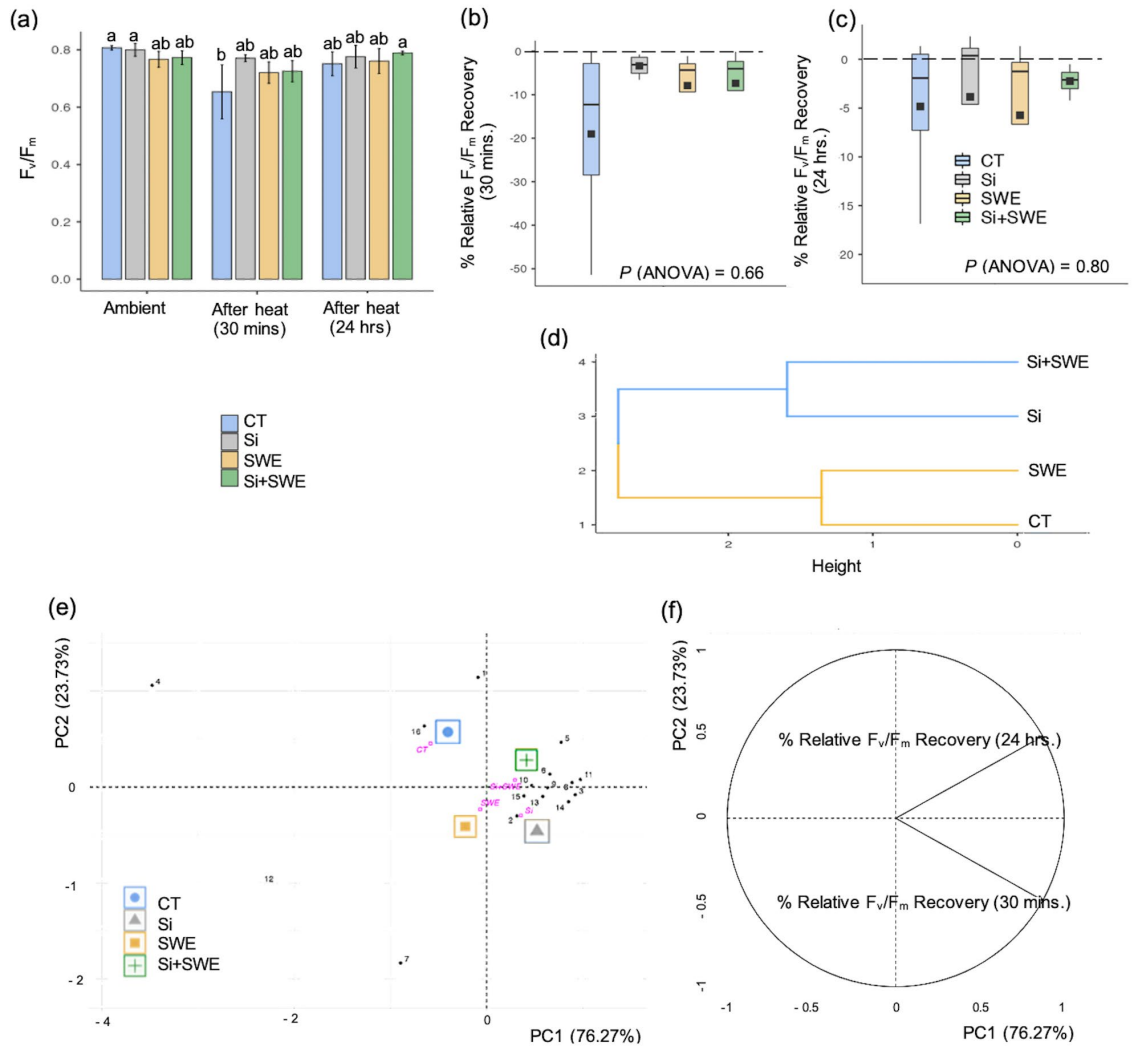
(**a**) Photosynthetic efficiency (F_v_/F_m_) in the control (CT), silicon (Si), seaweed extract (SWE), and Si plus SWE (Si + SWE) in the coffee plants under ambient conditions for 30 min and 24-h after heat. (**b**, **c**) Relative F_v_/F_m_ recovery 30 min and 24-h following heat treatment. (**d**) Cluster dendrogram of the F_v_/F_m_ recovery, and (**e**, **f**) PCA dividing influence of the relative F_v_/F_m_ recovery in the four treatments and the two periods. Different letters in each column represent significant differences (*p *≤ 0.05) using DMRT

Photosynthetic efficiency (F_v_/F_m_) in Si-treated, SWE, and Si + SWE plants showed a range of 0.72–0.78 after 30 min of heat treatment, compared to 0.65 in the control plant. The 24-hour recovery range was 0.76–0.78 (*p* ≤ 0.05) (Fig. 5a). The Si-treated plant had a higher relative F_v_/F_m_ zero-base-line recovery rate at -3.38, while in the SWE, Si + SWE, and CT treatments it was −7.89, −7.37, and − 19.02 (*p* = 0.66), respectively, over 30 min. The Si-treated and Si + SWE-treated plants recovered faster than the SWE and CT plants (−5.73 and − 4.83) at *p* = 0.80, especially over 24 h (Fig. 5c). The dendrogram also divided F_v_/F_m_ recovery into two groups: Si-treated and Si + SWE plants, and SWE and CT plants (Fig. 5d). PCA in the two recovery phases at correlation coefficients of 0.87 (*p* = 9.96e^-06^) showed that Si-treated Fv/Fm recovery, especially with SWE, may take longer (Fig. 5e, f).

**Fig. 6:**
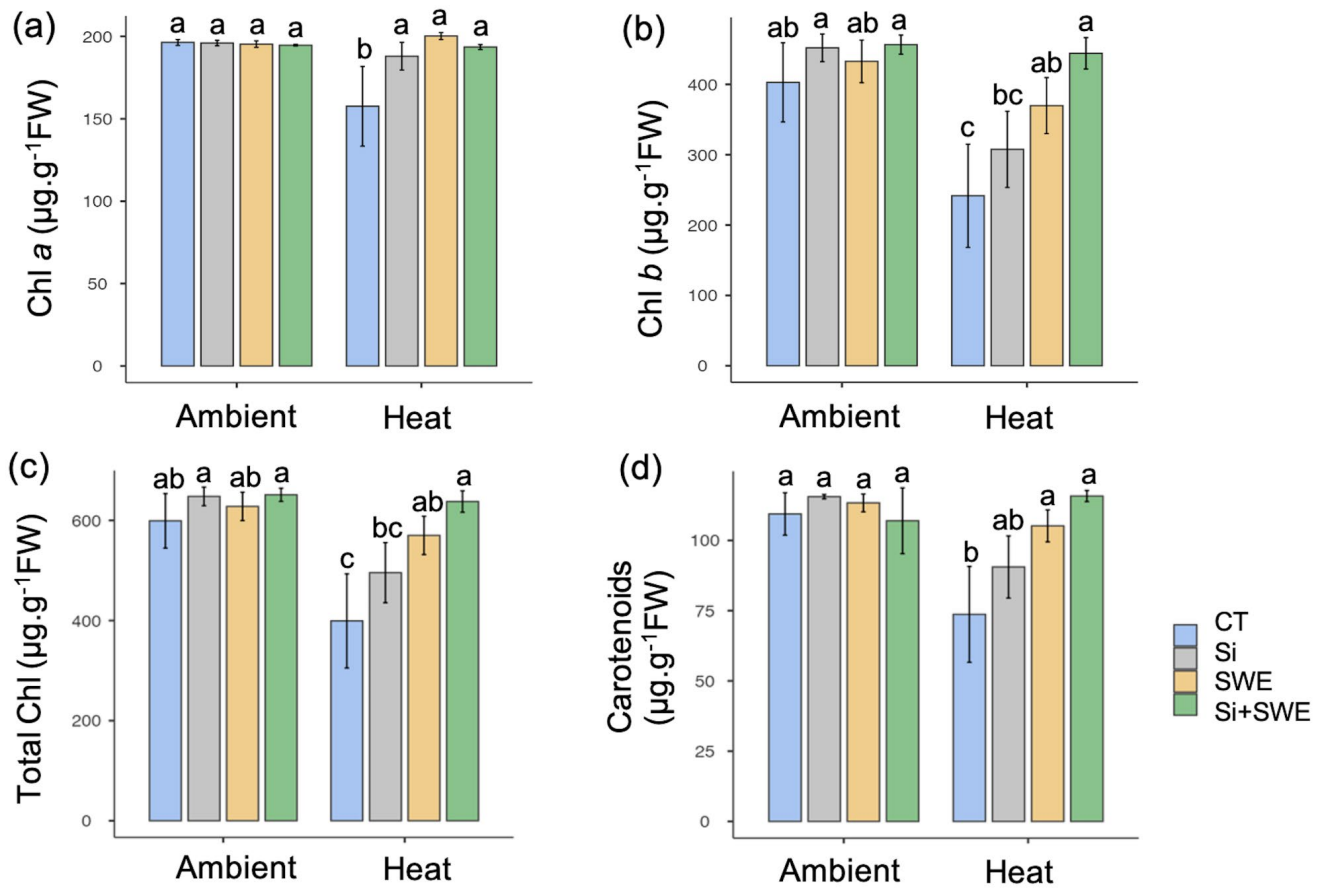
Contents of (**a**) chlorophyll *a*, (**b**) chlorophyll *b*, (**c**) total chlorophyll, and (**d**) carotenoids in the control (CT), silicon (Si), seaweed extract (SWE), and Si plus SWE (Si + SWE) treatments in the coffee plant under ambient and heat conditions. Different letters in each column represent significant differences (*p* ≤ 0.05) using DMRT

The Si + SWE and SWE plants had increased chlorophyll *a* (193.56 and 200.25 µg g^-1^ FW) and *b* (444.18 and 369.79 µg g^-1^ FW) levels following heat exposure compared to Si-treated and CT plants (Fig. 6a, b). Si + SWE showed considerably higher chlorophyll *a* and *b* levels (157.59 and 241.68 µg g^-1^ FW) in the heat compared to the CT plant (*p* ≤ 0.05). In heat conditions, chlorophyll *b* content was highly correlated with total chlorophyll content in the four treatment plants. In the heat, the Si + SWE treated plant had a considerably higher total chlorophyll content (570.04 µg g^-1^ FW) compared to the CT plant µg g^-1^ FW. Similar trends were observed in carotenoid concentrations under heat conditions (Fig. 6). The increase in chlorophyll and carotenoid content in the Si + SWE plant under the heat conditions (Fig. 6) seemed to represent the F_v_/F_m_ encouragement in the treated plants (Fig. 5).

**Fig. 7:**
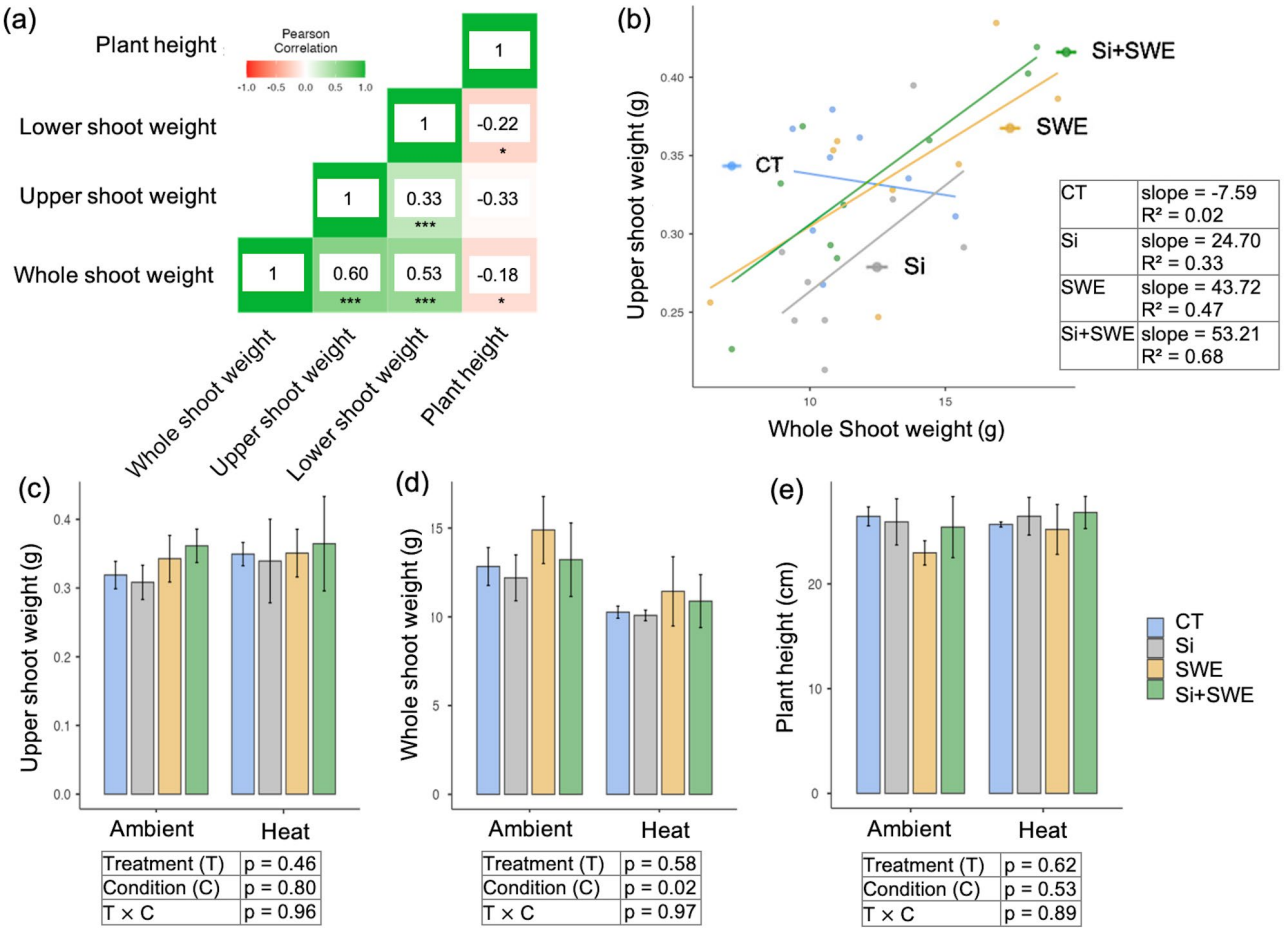
Changes in plant weight and height under (**a**) Pearson coefficient correlation and (**b**) linear correlation, and (**c-e**) comparison of upper and lower shoot weight, whole shoot weight, and plant height in the coffee plant in the control (CT), silicon (Si), seaweed extract (SWE), and Si plus SWE (Si + SWE) treatments under ambient and heat conditions

A stronger association (*r* = 0.61; *p* < 0.001) between the weight of the upper shoot and the whole shoot weight, with the basal shoot weight following closely behind (*r* = 0.53; *p* < 0.001). This association suggests that the shoot apex of coffee has developed slightly more than the basal shoot. The linear and bar graphs for the upper shoot weight and whole shoot weight showed that the Si + SWE treated plant (slope: 53.21) and the SWE treated plant (slope: 43.71) had a wider range of relation improvement than the Si plant (slope: 24.70), while the CT plants had a low correlation (slope: -7.59). (Fig. 7b, c). The plant height negatively correlated with these three shoot weight parts (Fig. 7a). This negative correlation may affect plant resilience under heat stress by emphasising weight gain in SWE and Si + SWE treated plants compared to Si and CT plants (Fig. 7c-e). The upper shoot weight and shoot height of coffee plants treated with Si + SWE were better than those treated with Si (at 24.13%, 1.36%) or SWE (at 2.85%, 6.42%) alone under heat conditions, respectively (Fig. 7c, e).

## Discussion

This study examined how nanosilicon and seaweed extract, which may promote element balance through homeostasis, affect photosynthetic performance in heat-stressed Arabica coffee plants. Specifically, the nanosilicon control was necessary to restore Arabica coffee plants’ photosynthetic efficiency (F_v_/F_m_), whereas the seaweed extract enhanced chlorophyll concentration. The two substances may improve Arabica coffee heat tolerance.

### Silicon homeostasis on coffee plant minerals under heat conditions

Si regulation differs by plant species; hence, its overall significance in respect to other elements is still unknown [14]. Most of the relatively variable PC-loading PC1 (74.4%) aligns with element ratios of Mg, P, Cl, K, Mn, Fe, Cu, and Zn per Si and affects the upper shoot under heat stress (Fig. 1). There is a scarcity of studies examining elemental aspects of Si homeostasis. Pavlovic et al. [14] suggested that Si may need the balance of Mg, P, K, Mn, Fe, and Zn, especially under nutrient deficiency stress. In this study, the Si-performance ratio was related to the promotion ratio of these eight components, particularly into the shoot apex of the coffee plant under heat stress. Si storage was enriched in the roots of Arabica coffee plants with the closest value scale 1 (Fig. 2). The dicotyledonous plants like tomatoes also absorb more Si from their roots [40]. Si is peculiarly placed within a group of Mg and Fe under heat and ambient circumstances (Fig. 2). Gunes et al. [41] found that external Si-regulated Mg and Fe changed in sunflower plants exposed to drought stress. The effects of Si in Arabica coffee plants may be best explained by the relationship between Mg and Fe. However, Si, SWE, and Si + SWE-treated coffee plants at the upper shoot had lower Fe ratios than Mg, especially in heat stress (Fig. 3). It corroborates Grege et al. [42], who found that spraying Si (K_2_SiO_3_) to wheat and carrot resulted in a rich accumulation of Fe in root tissues (~ 40%), despite only 10% Fe in shoots. The low shoot Fe concentration was caused by silicon, which formed a barrier in the cell to reduce Fe flux into the apoplastic space and higher Fe-chelating production, as shown in rice [43]. Grege et al. [42] also found that Fe enriches in the root tissues and prevents translocation to the shoot, resulting in a low TF value. Similarly, the examination of twelve wheat cultivars revealed that Fe collected in the root rather than the shoot [44]. This study revealed that Fe transmission (TF value from the root) under heat conditions was influenced more by the basal shoot section than the shoot apex (Fig. 4). Thus, Fe regulation under Si influence may not affect shoot Fe primary efficiency compared to Mg under heat conditions.

In contrast, Si treatment may increase Mg flow into the Arabica coffee upper shoot, especially under heated Si and Si + SWE treatments (Figs. 3 and 4). Si increased Mg in potted sunflower shoots compared to water stress alone [41]. Grege et al. [42] found that Si increased Mg mobility up via shoots rather than accumulating in roots in six plant species. Buchelt et al. [45] showed that Si could improve Mg usage efficiency rather than absorption to reduce Mg stress in Mg-deficient forage plant crops.

However, the mechanism of Mg mobility in the Si-Mg relationship under heat is still unknown. Apparently, Mg nutrient prefer mobility to shoot source as leaves to protect photosynthetic activity [46]. Mg modulation promotes thermal stability and catalytic activity of Rubisco activation, which enhances chlorophyll stability and photosynthetic performance under heat stress [47–50]. Previously, it has been found that adequate Mg prevented protein denaturation in coffee seedlings caused by heat stress (35 °C) [51].

Si has a greater effect on Mg efficiency in shoot than Fe; the outcomes may benefit from the Si-Mg relationship. For example, Si and Si + SWE-treated plants had 54.20 and 67.28% Mg/Si increases in the upper shoot compared to CT plants (Fig. 3). The Mg efficiency is predicted to react with photosynthetic defence against heat stress in Arabica coffee plants.

### Synergistic effects of Si and SWE on photosynthetic ability in the coffee plant under heat effect

In the excessive heat, chlorophyll concentrations and photosynthetic efficiency, F_v_/F_m_, were downregulated in the CT plant without supplements, at 24.36–66.67% and 7.07–19.18% lower than ambient CT, respectively (Figs. 5 and 6). Heat stress causes chlorophyll degradation [52], significant leaf photosystem II deflection, decreased F_v_/F_m_ under extreme heat (42 °C), and humidity shock in *Coffea canephora* [53]. Photochemical reactions caused cellular energy imbalance in chloroplast thylakoid lamellae, lowering variable fluorescence (F_v_/F_m_) [54]. These could be the result of heat-damaged chloroplast structures in leaf cells, as observed in a coffee study in Brazil [55].

Despite the heat, the photosynthetic efficiency, F_v_/F_m_, tended to keep recovery in a group treatment containing Si, Si plant, and Si + SWE treated plants from − 3.84 to -2.23 through 24 h, while the SWE plant recovered poorly (-5.73) (Fig. 5). Silicon supplementation may have increased F_v_/F_m_ in stressed plants by absorbed light allocation, as in drought-tolerant tomato [56, 57]. As with coffee, Si homeostasis should improve F_v_/F_m_ recovery and Mg modulation. El-Ezz et al. [58] further suggested that F_v_/F_m_ performance might accurately indicate plant Mg assessment. In the event of photosynthesis disruption, numerus functions of Mg maintained photosynthetic activity [46]. It implies the Si may prevent F_v_/F_m_ from disrupting Mg homeostasis in the present study.

SWE-treated plants had 6.52%, 20.20%, and 15.01% more chlorophyll *a*, *b*, and total content than Si-treated plants in heat stress. It implies that SWE protects coffee plant chlorophyll pigments from heat better than Si. Chlorophyll pigment in the leaf tissues of strawberry was similarly raised (by 2–12%) when treated with seaweed extract (TAM^®^) [17]. Ali et al. [16] suggested that SWE improved plant membrane nutrient transporters that increased chlorophyll and carotenoid contents. SWE also contained betaine, which prevented chlorophyll degradation and preserved photosynthetic efficiency in cucumbers [59], largely attributable to chloroplast biogenesis for horticultural plant improvement [60].

Si + SWE treatment increased chlorophyll pigments and F_v_/F_m_ more than SWE and Si alone under heat conditions. Si and SWE interactions may regulate Mg homeostasis, chlorophyll, and photosynthetic efficacy in the coffee upper shoot to minimise severe heat effect. This suggests Si and SWE synergistically affect coffee plants. The combination of seaweed extract (3 g/L) with nanosilicon (100 mg/L) increased chlorophyll pigments and F_v_/F_m_ in *Rosa damascena* in case of salt stress in vitro [61]. In the present study, the coffee plant with the Si + SWE supplement had 83.78% more chlorophyll *b* than the CT plant under heat (Fig. 6). The expansion of the antenna’s absorption spectrum was shown to be significantly influenced by the structure of chlorophyll *b* in the photosystem [62]. The dominant chlorophyll *b* in photosystem II was damaged by extreme heat, impeding electron transport [63, 64]. High chlorophyll *b* levels in the Si + SWE may assist in repairing the photosystem under heat stress in the coffee plant.

The exogenous application of Si + SWE encouraged element homeostasis in photosynthetic apparatus, particularly chlorophyll *b*, and photosynthetic efficiency (F_v_/F_m_), involving the Mg balance, reflecting the remaining to the maintenance of shoot growth in the Arabica coffee plant under heat stress.

## Conclusions

The Arabica coffee plant is highly heat-sensitive. In this study, silicon promoted element ratio (per silicon) in coffee shoots under heat conditions. In response to heat, the Si homeostasis in the upper shoot of the coffee plant balanced elements, particularly Mg. Silicon management improved coffee plants’ photosynthetic efficiency (F_v_/F_m_), while seaweed extract increased chlorophyll. At 49 °C, Si + SWE supplementation increased photosynthetic pigments and F_v_/F_m_ in coffee plants, occasionally affecting Mg element homeostasis in the upper shoot. Si and SWE synergy could be used in agricultural operations to improve crop resilience for high-temperature coffee cultivation. Future research will focus on Si-SWE stability mechanisms for long-term heat effects.

## Electronic supplementary material

Below is the link to the electronic supplementary material.

Supplementary Material 1

## Data Availability

The data shall be available on reasonable request to SY.
