# Supplementary material for: Mitigating excessive heat in Arabica coffee using nanosilicon and seaweed extract to enhance element homeostasis and photosynthetic recovery
Source: BMC Plant Biol. 2024 Nov 12;24:1064. doi: 10.1186/s12870-024-05784-0 (PMC11555975; doi:10.1186/s12870-024-05784-0)
Supplement: Supplementary file 1 — Supplementary Material 1 [file 12870_2024_5784_MOESM1_ESM.docx]

**Supplementary**

**Mitigating excessive heat in Arabica coffee using nanosilicon and seaweed extract to enhance element homeostasis and photosynthetic recovery**

Ekkachak Chandon^1^, Patchawee Nualkhao^1^, Metee Vibulkeaw^1^, Rujira Tisarum^2^, Thapanee Samphumphuang^2^, Jianqiang Sun^3^, Suriyan Cha-um^2^, and Suravoot Yooyongwech^1*^

^1^School of Interdisciplinary Studies (Kanchanaburi Campus), Mahidol University, Kanchanaburi 71150, Thailand

^2^National Center for Genetic Engineering and Biotechnology (BIOTEC), National Science and Technology Development Agency (NSTDA), Pathum Thani 12120, Thailand.

^3^Research Center for Agricultural Information Technology, National Agriculture and Food Research Organization, 3-1-1 Kannondai, Tsukuba, Ibaraki 305-8517, Japan

**Table S1.** Information of the seaweed extract.

| Appearance | Black Brown Powder |
| --- | --- |
| Organic matter | 40 - 50 % |
| pH | 8 - 10 |
| Specific gravity | 0.1 - 0.2 g /cm^3^ |
| Total Nitrogen: | 0.5 % |
| Phosphorus (P_2_O_5_) | 3.0 % |
| Potassium (K_2_O) | 20 - 22 % |
| Amino acid | 4 % |
| Mannitol | 3 % |
| Mg | 0.06 % |
| Ca | 0.4 -1.6 % |
| Fe | 0.15 - 0.3 % |
| Cu | 25 - 45 ppm |
| S | 1.0 - 1.5 % |
| I | 300 – 600 ppm |

**Table S2**. Information of correlation coefficients

| Correlation in pairs of silicon and target element (magnesium and iron) contents in ambient condition | | | | | | |
| --- | --- | --- | --- | --- | --- | --- |
|  | |  | | Si_Am | |  |
| Si_Am |  | Pearson's r |  | — |  |  |
|  |  | p-value |  | — |  |  |
| Mg_Am |  | Pearson's r |  | 0.940 | *** |  |
|  |  | p-value |  | < .001 |  |  |
| Fe_Am |  | Pearson's r |  | 0.950 | *** |  |
|  |  | p-value |  | < .001 |  |  |
| Note. * p < .05, ** p < .01, *** p < .001 | | | | | | |
|  | | | | | | |

| Correlation in pairs of silicon and target element (magnesium and iron) contents in heat condition | | | | | | |
| --- | --- | --- | --- | --- | --- | --- |
|  | |  | | Si_Ht | |  |
| Si_Ht |  | Pearson's r |  | — |  |  |
|  |  | p-value |  | — |  |  |
| Mg_Ht |  | Pearson's r |  | 0.942 | *** |  |
|  |  | p-value |  | < .001 |  |  |
| Fe_Ht |  | Pearson's r |  | 0.973 | *** |  |
|  |  | p-value |  | < .001 |  |  |
| Note. * p < .05, ** p < .01, *** p < .001 | | | | | | |
|  | | | | | | |
